# STEM/Non-STEM Divide Structures Undergraduate Beliefs About Gender and Talent in Academia

**DOI:** 10.3389/fsoc.2019.00026

**Published:** 2019-04-12

**Authors:** Kimberlyn A. Bailey, David Horacek, Steven Worthington, Ampalavanar Nanthakumar, Scott Preston, Carolina C. Ilie

**Affiliations:** ^1^Department of Philosophy, State University of New York at Oswego, Oswego, NY, United States; ^2^Institute for Quantitative Social Science, Harvard University, Cambridge, MA, United States; ^3^Department of Mathematics, State University of New York at Oswego, Oswego, NY, United States; ^4^Department of Physics, State University of New York at Oswego, Oswego, NY, United States

**Keywords:** gender bias, women in science, underrepresentation of women, talent, STEM/non-STEM divide, gender stereotypes, STEM

## Abstract

Research and popular debate on female underrepresentation in academia has focused on STEM fields. But recent work has offered a unifying explanation for gender representation across the STEM/non-STEM divide. This proposed explanation, called the field-specific ability beliefs (FAB) hypothesis, postulates that, in combination with pervasive stereotypes that link men but not women with intellectual talent, academics perpetuate female underrepresentation by transmitting to students in earlier stages of education their beliefs about how much intellectual talent is required for success in each academic field. This theory was supported by a nationwide survey of U.S. academics that showed both STEM and non-STEM fields with fewer women are also the fields that academics believe require more brilliance. We test this top-down schema with a nationwide survey of U.S. undergraduates, assessing the extent to which undergraduate beliefs about talent in academia mirror those of academics. We find no evidence that academics transmit their beliefs to undergraduates. We also use a second survey “identical to the first but with each field's gender ratio provided as added information” to explicitly test the relationship between undergraduate beliefs about gender and talent in academia. The results for this second survey suggest that the extent to which undergraduates rate brilliance as essential to success in an academic field is highly sensitive to this added information for non-STEM fields, but not STEM fields. Overall, our study offers evidence that, contrary to FAB hypothesis, the STEM/non-STEM divide principally shapes undergraduate beliefs about both gender and talent in academia.

## 1. Introduction

Established stereotypes linking men but not women with innate brilliance may hinder women's paths into academia (Bennett, 1996; Tiedemann, 2000; Kirkcaldy et al., 2007; Lecklider, 2013; Leslie et al., 2015). Indeed, a 2015 study by Leslie et al. found that academic disciplines whose members highly value unteachable talent have gender ratios skewed toward men at the doctoral level. Using a nationwide survey of postdoctoral researchers, faculty and graduate students (henceforth: academics) in 30 disciplines, the authors found that the disciplines with the fewest women had practitioners who most strongly considered talent essential to success in their field (Leslie et al., 2015). Based on these findings, Leslie et al. proposed a theory for female underrepresentation in academia: the field-specific ability beliefs hypothesis. A field-specific ability belief (FAB) is the extent to which one believes that success in a given academic field requires talent. The authors propose that FABs are passed down from academics, saturating the general public, and combine with stereotypes about women's intellect to create and perpetuate the academic gender gap.

Leslie et al. build on prior research that suggests people vary in the extent to which they believe unteachable, fixed talent is essential to success in any activity. The FAB hypothesis is grounded in Carol Dweck's work on the “growth” vs. “fixed” mindset. Dweck's work suggests that individuals may be placed on a spectrum, where, on one end, an individual believes talent is innate (“fixed”) and, on the other end, an individual believes talent may be cultivated through effort (“growth”) (Dweck, 2006). The FAB hypothesis builds on Dweck's distinction, proposing that, rather than focusing on placing people along that “growth” vs. “fixed” spectrum, entire academic fields may be placed along that same spectrum. On this spectrum—so Leslie et al. suggest—success in some academic fields is widely believed to require “fixed” unteachable intellectual talent, while success in other academic fields is widely believed to require hard work (Leslie et al., 2015). They propose that the degree to which academics believe success in a given academic field requires fixed talent – their FABs – strongly influences the extent to which the wider public believes a given academic field requires fixed talent.

The FAB hypothesis also includes specific claims about how FABs play into causal mechanisms responsible for the gender gap. Key to understanding their proposed mechanism is that field-specific ability beliefs are a metric of the extent to which an academic field is believed to require fixed, unteachable brilliance, as opposed to talent that can be developed through hard work. If a field is indeed believed to require unteachable brilliance, then, naturally, success in that field becomes viewed as an insurmountable challenge for anyone who feels he or she lacks that innate intellectual spark. The FAB hypothesis posits, first, that academics hold negative stereotypes about women's innate intellectual ability and thus exhibit biases against them in high-FAB fields (Valian, 1999). The FAB hypothesis posits, second, that women internalize these stereotypes about themselves and/or believe that such pervasive stereotypes render high-FAB fields inhospitable to them and, as a result, decide to not pursue high-FAB fields (Wigfield and Eccles, 2000; Dar-Nimrod and Heine, 2006).

For such mechanisms to explain the gender gap, young women making decisions about entering academia must be able to reliably identify high-FAB “brilliance required” fields as such. This is an explicit prediction of what, in a later follow-up study by the same research group, is called the “extended FAB hypothesis” (Meyer et al., 2015). Within this extended framework, the authors posit that those with exposure to a given academic field will “absorb” the FABs of the practitioners of that discipline (Meyer et al., 2015). It is this tenet that we aim to test. If true, then we should expect undergraduate FABs to strongly reflect those of academics or come to do so as undergraduates spend more time in college. The researchers propose that their “extended FAB hypothesis” includes the general public, such that the public at large holds FABs like those of academics. Using another survey-based study, they found this to be the case and, further, that those with college-level exposure to a field have FABs that more closely reflect those of academics than those without (Meyer et al., 2015). Insofar as undergraduates have constant and direct exposure to academics, they are the population that seems most likely to “absorb” the FABs of academics. Indeed, studies show that the largest drop-off of women in male-dominated fields happens at the undergraduate level (Ceci et al., 2014). It is also predominantly undergraduates who face the choice about whether to attend graduate school, and if they decide to go, which field to enter. If the FAB hypothesis is to explain their academic choices at this crucial juncture, and the gender gap more generally, then undergraduate FABs must become aligned with those of the academics with whom they are in constant contact. We test this top-down inheritance of beliefs using a national survey of undergraduates that mirrors the one used by Leslie et al. to estimate undergraduate FABs and compare these to the FAB scores of academics collected by Leslie et al.

We test an additional key prediction of the FAB hypothesis about the similarity of undergraduate and academic FABs. Current research about the gender gap in academia has largely focused on women's underrepresentation in STEM (Ceci and Williams, 2007). Yet there is considerable variation in female representation on both sides of the STEM/non-STEM divide (Ceci and Williams, 2007, 2011). Less than 20% of all physics Ph.D.'s are awarded to women, while neuroscience programs award around 50% (National Science Foundation, 2011). Similarly, women currently earn more than 70% of all Ph.D.'s in art history, but less than 35% in philosophy (National Science Foundation, 2011). Leslie et al.'s FAB hypothesis is novel in that it offers a unified explanation for variation in female representation across the STEM/non-STEM divide. Indeed, the FABs of academics were predictive of female representation in academia both across the STEM/non-STEM divide and within these subsets of fields. Thus, the FAB hypothesis postulates that undergraduate FABs should be predictive of female representation not only within STEM fields, but also non-STEM fields and across both subsets of fields combined.

As a second part of our study, we also seek to explicitly explore the relationship between undergraduate beliefs about gender and talent. We also use a second survey that, like the first, asks undergraduates to rate the extent to which they believe a given academic field requires talent, with one key difference: The gender ratio for each academic field is given as added information.

Overall, our study serves as a test of some key predictions of the FAB hypothesis about undergraduate beliefs and, more generally, as a study into undergraduate beliefs about gender and talent in academia.

### 1.1. Summary of Predictions

We sought to test several key predictions of the FAB hypothesis using survey version one (respondents not given the female representation of each field as added information). We thus formed our hypotheses for survey one in accordance to what the FAB hypothesis would predict. In this section we briefly summarize our hypotheses, with more details furnished in the introduction and discussion sections. Just as Leslie et al.'s original study, we used the percent of Ph.D.'s awarded in 2011 within the US to females and African Americans as our metric for female and African American representation in academia, respectively (National Science Foundation, 2011).

The FAB hypothesis claims that the FABs of undergraduates are strongly influenced by the FABs of academics and, thus, the hypothesis predicts that undergraduate FABs, like those of academics, predict female representation. We thus hypothesized that, (1) as Leslie et al. found for academic FABs, there would be an association between average undergraduate FABs for each field and female representation for each field.

The FAB hypothesis claims that FABs play an important explanatory role for female representation in academia across all academic fields. Thus, the FAB hypothesis posits that FABs are predictive of female representation not only for STEM fields, but also non-STEM fields. We hypothesized that, as Leslie et al. found for academic FABs, (2) there would be an association between average undergraduate FABs and female representation for STEM fields alone, non-STEM fields alone and across all fields and (3) undergraduate FABs would remain an important predictor of female representation, even when a field's classification as STEM or non-STEM is taken into account.

The FAB hypothesis claims that FABs, as beliefs about how much innate talent is required for a given academic field, influence representation in academia of any group stereotyped as lacking innate intellectual talent. African Americans are one such group (Steele and Aronson, 1995). We thus hypothesized that, (4) as Leslie et al. found for academic FABs, there would be an association between undergraduate FABs and African American representation.

The FAB hypothesis claims that the FABs of academics influence the FABs of the public at large, such that those with more exposure to academics develop FABs that more closely resemble those of academics. Thus, the FAB hypothesis predicts that undergraduates—who have direct and constant exposure to academics—will have FABs that resemble those of academics or come to do so with more exposure to academics. We thus hypothesized that (5) undergraduate FABs would differ between undergraduate class years (Freshman, Sophomore, Junior or Senior) as a result of differing degrees of exposure to academics.

With survey version two (respondents given the female representation of each academic field as added information), we sought to generally probe the relationship between undergraduate beliefs about gender and talent in academia and to test a specific implication of the FAB hypothesis. The FAB hypothesis builds off of work that suggests are stereotyped as having less innate intellectual talent than men. Informing undergraduates of the gender ratio in each academic field could thus be expected to trigger those stereotypes and strongly influence respondent FABs. The FAB hypothesis thus predicts that (6) respondents provided the gender ratio in each academic field should give FABs that, relative to undergraduates not provided that added information, rank male-dominated fields as more “brilliance-required” fields (higher FABs) and female-dominated fields as less “brilliance-required” (lower FABs).

## 2. Methods and Materials

### 2.1. Survey Respondents and Administration

The FAB scores of academics—graduate students, postdoctoral students and faculty—were used from Leslie et al.'s original study (Leslie et al., 2015). To compare the FABs of academics and undergraduates, we surveyed undergraduates in this study.

Participants were composed of 1075 U.S. undergraduates from 197 universities across the Country, ranging from the Ivy League to community colleges. The surveys were approved by the Human Subjects Committee of State University of New York (SUNY) at Oswego. Data was excluded from an additional 586 individuals who had identified as living outside the US, not currently matriculated in an undergraduate university or who had failed to complete the survey within, at most, a few missing answers.

The majority of participants came from Le Moyne College in Syracuse, New York (24%) and SUNY Oswego (53%). The other 33% of participants were undergraduates who learned of the survey through Tumblr, a blogging site popular with undergraduates. We administered surveys to SUNY Oswego students during the spring 2015 semester. The classes in which surveys were administered covered a wide range of subjects from both STEM and non-STEM fields. While most SUNY Oswego students took a printed version survey during a class session, several larger classes at SUNY Oswego took an online version, via a link sent by their professors. The online version mirrored the paper version and was created using the website Survey Monkey. Le Moyne College students were prompted to complete the online version, linked in a school-wide email. Le Moyne students and Tumblr readers were incentivized to take the survey with entrance into a drawing for an $80 Apple gift card. A popular blogger on Tumblr initially posted an invitation to take the survey, and other bloggers re-posted or “reblogged” the original post, spreading the invitation to a wider audience. To ensure only the intended participants took the survey, the online version had an added question at the start of the survey: Participants were asked to confirm that they are currently enrolled undergraduates at an American university or college. Those who answered negatively were thanked for their time on the following screen and were not advanced to the next portion of the survey.

Prior to starting the survey, each respondent was informed that the purpose of the study is to examine undergraduate attitudes about academic fields. Additionally, respondents were informed that the study was approved by the Human Subjects Committee of SUNY Oswego and were asked for their participation for the sake of advancing social science.

### 2.2. Academic Fields

A total of 42 academic fields were included in the surveys. We used three criteria in choosing these fields: overlap with the fields included in the survey used by Leslie et al., the relative size of each field (by Ph.D.'s granted in 2011 National Science Foundation, 2011) and whether an average undergraduate could be expected to meaningfully distinguish the fields from one another. We kept 29 of the 30 fields used by Leslie et. al. rejecting only Comparative Literature on the grounds that the average undergraduate could not be expected to find it meaningfully distinguishable from English Literature, a field which we retained from the group of fields used in Leslie et al.'s survey.

We added an additional 13 fields from a diverse set of disciplines within both STEM and non-STEM fields, with broad variations in female representation. To ensure a relatively objective method for choosing fields and increase the likelihood that undergraduates would be familiar with the chosen fields, all of the 13 added fields were amongst those that produced the largest number of Ph.D.'s in 2011 (National Science Foundation, 2011). We did not include some of those fields on distinguishability grounds. For example, Curriculum and Instruction was excluded due to anticipated difficulties for the average undergraduate to meaningfully distinguish it from Education, which was already included from the Leslie et al.'s group of fields.

### 2.3. Field-Specific Ability Beliefs Survey Questions

To assess field-specific ability beliefs, participants were asked to rate their agreement with a statement, for each of the 42 fields. The statement was taken directly from Leslie et al.'s original survey of academics (Leslie et al., 2015) and reads, “Being a top scholar in this discipline requires a special aptitude that just can't be taught.” Participants rated their agreement on a 10-point scale, which was then converted to a seven-point scale, to match Leslie et al.'s scale.

Participants were randomly assigned to take one of two versions of the survey. In the first version, respondents rated their agreement with the above statement. In the second version of the survey, participants were asked to do the same, but were provided additional information: below the name of each field appeared a percentage bar representing the percent female representation within that field. The same measure used for female representation throughout our analyses was used in the survey: the percent of female Ph.D. recipients for a field in 2011 (National Science Foundation, 2011).

Respondents were told what the percentage bars represent and the source of data. Of all surveys retained for analysis, each of the two surveys was taken by roughly half of participants: 546 participants (40% male, 47% STEM majors) and 518 (37% male, 24% STEM majors) participants took survey version one and two, respectively. Respondents who took the online version of the survey were randomly assigned a survey version. For respondents who took an in-class survey, each class was administered the same survey, but which classes received which version was randomly assigned.

### 2.4. Education and Demographic Questions

For both versions of the survey, participants were asked a number of questions pertaining to their educational and demographic characteristics. They were asked to report their major(s), future plans after graduation (or to indicate that they did not know, if such was the case), grade point average (GPA), gender, race, class year, and university. Major(s), future plans, GPA, and university were open ended questions, while race (white, black, or African American, American Indian or Alaskan native, Asian American, native Hawaiian, or other Pacific Islander, Latino/Hispanic, middle eastern), gender (female, male or other) and class year (freshman, sophomore, junior, or senior) were multiple choice. Based on the university of the respondent, we also looked up the average American College Test (ACT) score of accepted freshmen to his or her university from collegedata.com and recorded the number for each respondent. This acted as a rough measure of the prestige of his or her university.

To ensure categorical variables with open ended responses in the survey could be usefully parsed and analyzed, responses were coded in a simplified fashion. Major(s) were categorized as STEM, non-STEM or both (when a respondent had double or triple majors that included both STEM and non-STEM). Future plans were coded with attention to the central concerns of this study: the field into which one goes and how far one plans to advance into said field. Future plans after graduation were classified based on the field the respondent intended to pursue, if known, and in what capacity he or she wanted to go into the field “not necessarily academic. A respondent planning to get a Ph.D. in Chemical Engineering, for example, was classified as G-STEM for graduate studies in STEM, while a respondent intending to work in social services was classified as J-SS for job in social services. A full list of questions in the survey and coding schemes are outlined in Table S1.

### 2.5. Data Analyses

Below we discuss our hypothesis-driven analyses, which are numbered to match the hypotheses outlined in the introduction. Additional analyses are described in the results section.

Our study was designed primarily as test of whether undergraduate FABs mirrored those of academics. Toward that end, we replicated several statistical tests used on academic FABs collected by Leslie et al., using, instead, the undergraduate FABs we collected. (1) To test whether undergraduate FABs were, like academic FABs, strongly predictive of female representation, we used Pearson correlations between the average undergraduate FAB for each field and female representation for each field. (2) To test whether undergraduate FABs were, like those of academics, predictive of female representation across all fields, as well as STEM and non-STEM fields separately, we computed Pearson correlations for all fields, for STEM fields and for non-STEM fields. (3) Leslie et al. found that a field's classification as STEM or non-STEM became an unimportant predictor for female representation when academic FABs were added in a stepwise hierarchical regression. To test whether the same was true for undergraduates, we replicated the same analysis. We used a stepwise hierarchical regression with, first, a field's classification as STEM or non-STEM as a predictor for female representation in each field, and second, average undergraduate FAB for each field as an additional predictor. (4) Leslie et al. found that academic FABs were negatively correlated with African American representation for each field. To test whether the same was true with undergraduates, we used a Pearson correlation between average undergraduate FAB score and African American representation.

Our study was also designed to test other several key predictions of the FAB hypothesis. (5) The FAB hypothesis posits that academic FABs influence the FABs of the public. Undergraduates, in constant contact with academics, would thus be expected to have FABs that approach those of academics as they gain more college experience. To test this, we used a two-way ANOVA “with academic field and undergraduate FABs as predictors for female representation” to examine the effect the academic field and the effect that a student's class year (freshman, sophomore, junior or senior) had on undergraduate FAB scores. (6) The FAB hypothesis takes as a working premise that the public has internalized stereotypes that associate men, but not women, with innate brilliance. The FAB hypothesis thus predicts a key difference between the results for survey version one (undergraduates not provided gender ratios) and version two (undergraduates provided gender ratio): That undergraduates informed of the gender ratio within each academic field will, by virtue of these internalized stereotypes, tend to have FABs that are more predictive of female representation relative to undergraduates not informed. To test this, we computed Pearson correlations between average undergraduate FAB and female representation for each field using survey two data and compared these correlations to those computed for survey one.

## 3. Results

### 3.1. Hypothesis-Driven Results

We formulated several hypotheses that the FAB hypothesis would predict to be true of undergraduate FABs (see ending section of introduction). We found evidence for hypothesis (1) and found no evidence for hypotheses (2)–(6). In this section, we report the results for those predictions, indicating the number of the corresponding prediction in the text. We also report results for other analyses we used to explore our data. Although this second set of analyses were not hypothesis-driven, insofar as we did not find evidence for most of our hypotheses, we did these additional analyses to generally explore what conclusions our data could suggest.

**Figure 1:**
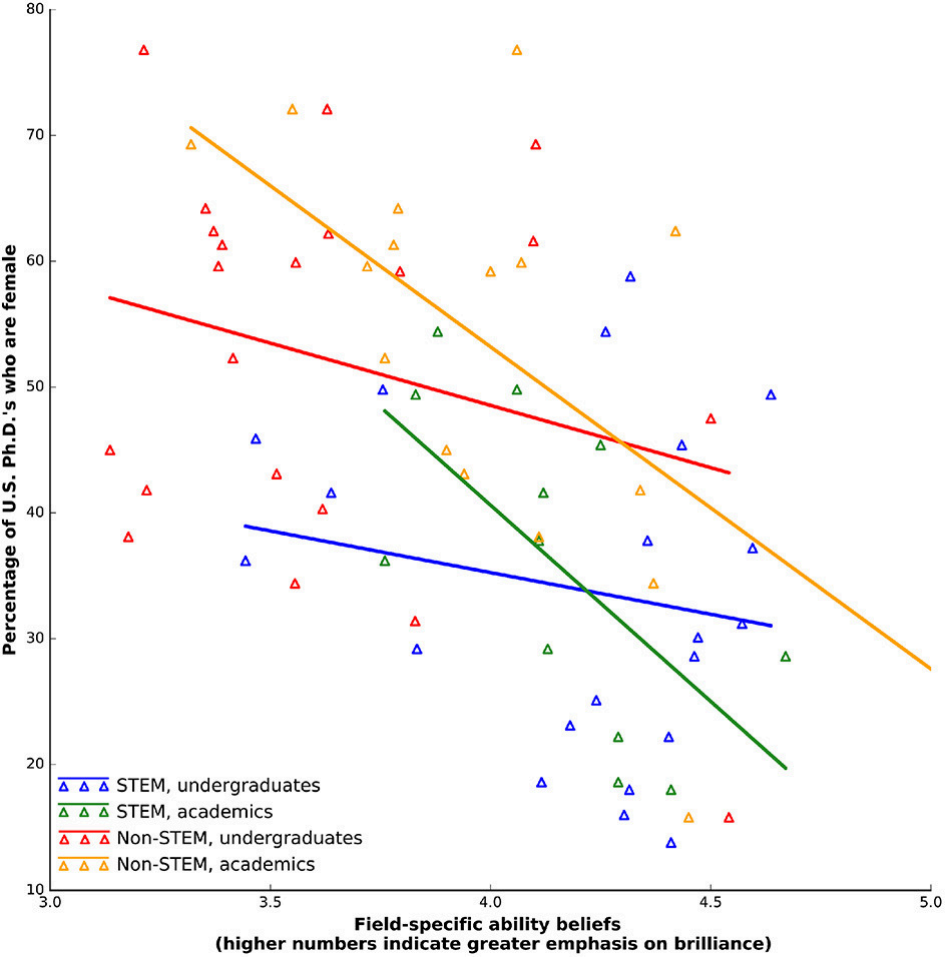
Undergraduate (survey excludes gender ratios) and academic field-specific ability beliefs vs. the percentage of female 2011 U.S. Ph.D.'s.

We did not find evidence to suggest that undergraduate FABs predict female representation. In turn, because academic FABs strongly predict female representation, we found no evidence to suggest that undergraduate FABs strongly resemble those of academics. (1) As the FAB hypothesis would predict, across all 42 fields, undergraduate FAB scores were, like those of academics, negatively correlated with female representation (Figure 1). (2) However, when we analyzed STEM and non-STEM fields separately, we did not detect any correlation for both STEM and non-STEM fields (Table 1).

**Table 1:** Pearson correlations between field-specific ability beliefs and African American and female representation, stratified by field type (STEM, non-STEM, all fields).

**Correlation**	**Field type**	**r**	***p***	**95% confidence interval**
**UNDERGRADUATES (SURVEY INCLUDES GENDER RATIOS)**				
FABs, percent female Ph.D.s	STEM	–0.31	0.20	–0.67, 0.17
	Non-STEM	0.68	0.002	0.25, 0.82
	All fields	0.28	0.08	–0.03, 0.54
**UNDERGRADUATES (SURVEY EXCLUDES GENDER RATIOS)**				
FABs, percent female Ph.D.s	STEM	–0.13	0.60	–0.55, 0.35
	Non-STEM	–0.10	0.69	–0.52, 0.36
	All fields	–0.47	0.002	–0.68, –0.19
FAB, percent African American Ph.D.s	All fields	–0.01	0.94	–0.31, 0.29
**ACADEMICS**				
FABs, percent female Ph.D.s	STEM	–0.64	0.03	–0.88, –0.10
	Non-STEM	–0.65	0.01	–0.86, –0.24
	All fields	–0.63	< 0.001	–0.81, –0.34
FAB, percent African American Ph.D.s	All fields	–0.53	0.002	–0.75, –0.21

*Results shown for academics (Leslie et al., 2015), as well as for undergraduates with and without gender ratios provided in the survey*.

A key finding of Leslie et al.'s study on academics was that when academic FABs are taken into account, the STEM/non-STEM divide becomes unimportant for predicting female representation, suggesting that the FAB hypothesis plays an important explanatory role for the gender gap independent of this divide (Leslie et al., 2015). (3) We replicated the stepwise hierarchical regression used by Leslie et al., using, instead, our undergraduate FABs. For academics, the STEM/non-STEM indicator were a non-significant predictor when academic FABs were added. For undergraduates, on the other hand, the STEM/non-STEM indicator remained significant when FAB scores were added and also mitigated the effect of those scores to the extent that they were a non-significant predictor (Table 2).

**Table 2:** Hierarchical regression models predicting female representation from undergraduate data (*n* = 42 academic fields).

	**Model 1**			**Model 2**		
**Predictor**	**$\hat{\beta }$**	**t**	***p***	**$\hat{\beta }$**	**t**	***p***
STEM/non-STEM categorization of field	−0.56	−4.26	<0.001	−0.42	−2.43	0.02
Undergraduate field-specific ability beliefs				–0.21	−1.20	0.24
Adjusted R^2^		0.295			0.303	
F statistic for change in adjusted R^2^		18.15			9.90	
*P*-value for change in adjusted R^2^		< 0.001			< 0.001	

(4) The FAB hypothesis predicts that the representation of populations who are, like women, stereotyped as lacking innate brilliance will be negatively correlated with FABs (Leslie et al., 2015). African Americans are one such group (Steele and Aronson, 1995). Leslie et al. found that academic FABs were negatively correlated with African American representation. We did not find evidence for the same with undergraduate FABs (Table 1).

(5) If academics pass on their FABs to undergraduates, increased exposure to academics should cause undergraduate FABs to converge with academic FABs. In a two-way ANOVA, we found no evidence that class year (freshman, sophomore, junior or senior) [*F*_(1, 22413)_ = 0.95, *p* = 0.33] or its interaction with academic field [*F*_(41, 22413)_, *p* = 0.87] had an effect on FAB scores. As a group, we found no evidence that undergraduate's FABs change during college, and, by extension, we found no evidence that they change through prolonged exposure to academics.

**Figure 2:**
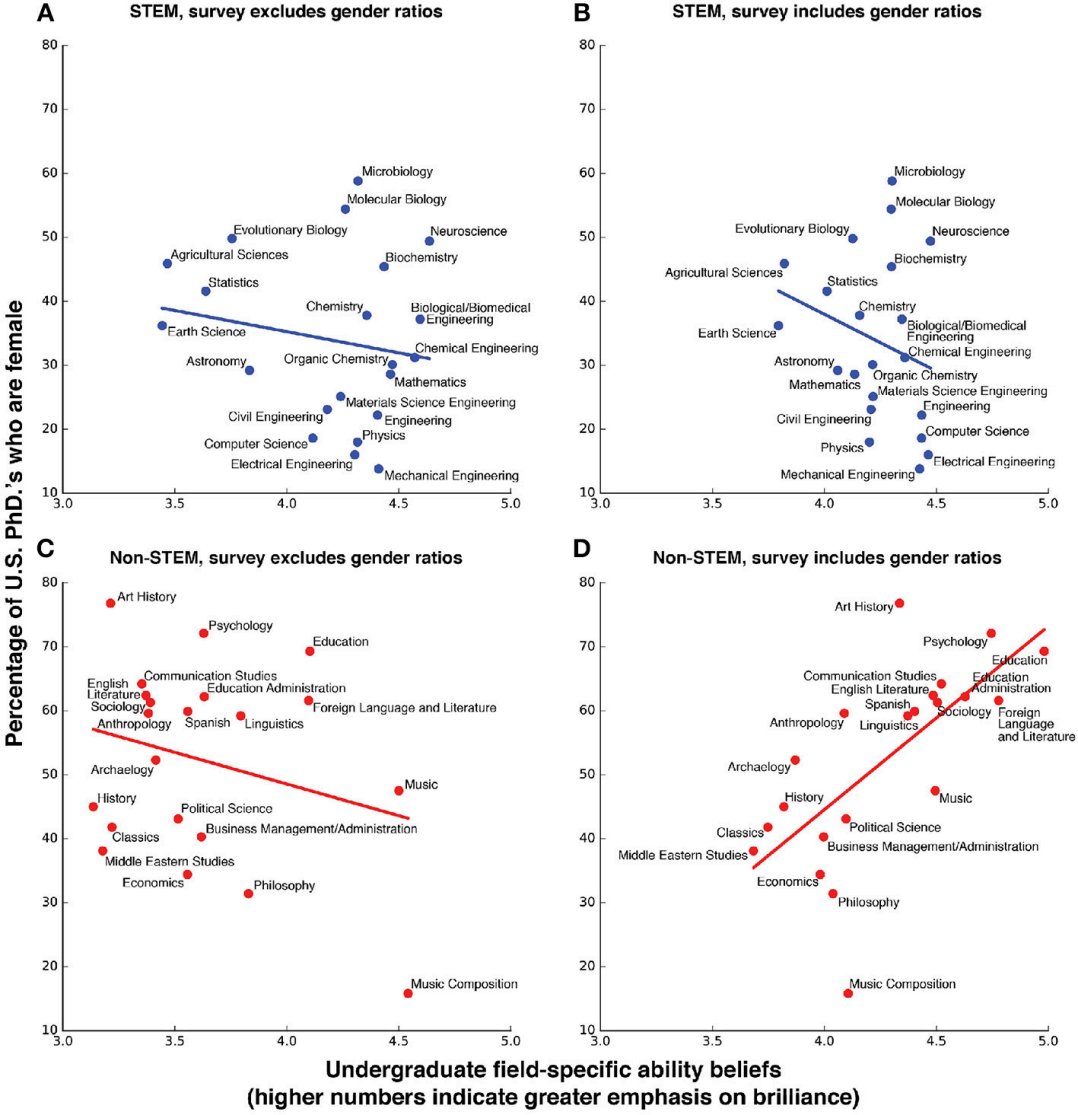
Undergraduate field-specific ability beliefs vs. female representation, with and without field-specific gen- der ratio information provided. Field-specific ability beliefs and the percentage of U.S. female Ph.D.'s in 2011 for STEM fields **(A)** without gender ratios included in the survey and **(B)** with gender ratios and non-STEM fields **(C)** without gender ratios included in the survey and **(D)** with gender ratios.

**Figure 3:**
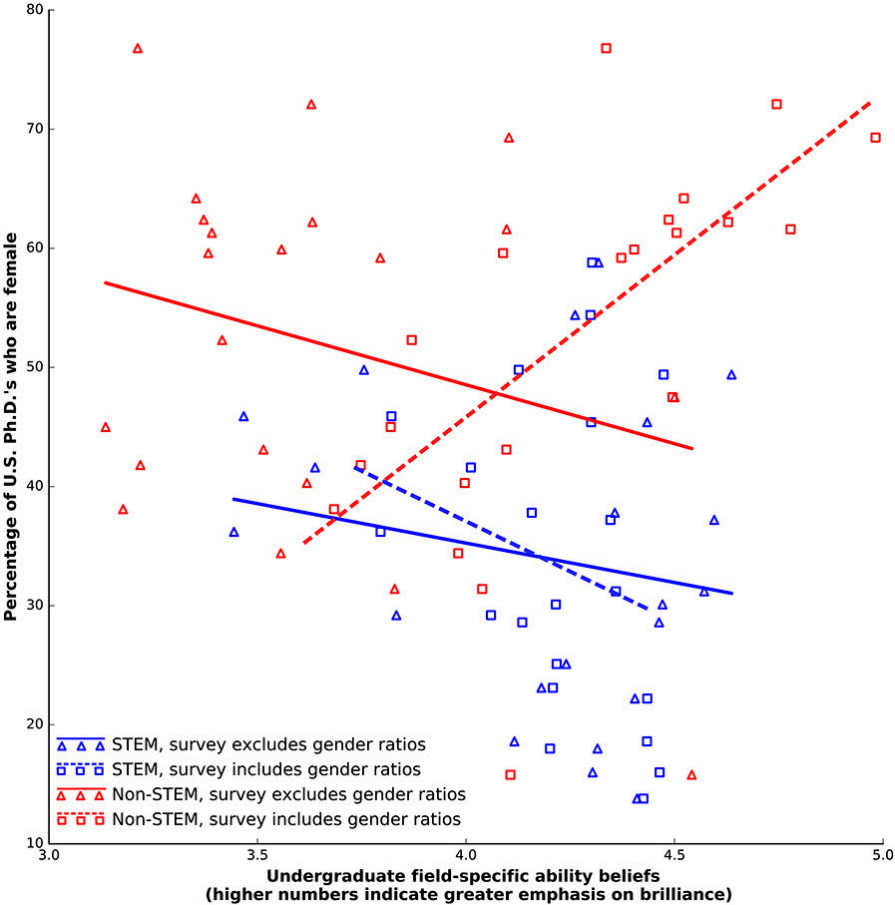
Undergraduate field-specific ability beliefs vs. the percentage of female 2011 US Ph.D.'s in (blue) STEM and (red) Social Sciences/Humanities fields, for both survey that excluded gender ratios (solid trend lines) and included the gender ratios (dotted trend lines).

Survey version two, for which undergraduates were provided the gender ratio in each academic field, provided no support for our last hypothesis. The FAB hypothesis suggests that (6) informing undergraduates of the gender ratio of each filed should give FABs that rank male-dominated fields as more “brilliance-required”fields (higher FABs) and female-dominated fields as less “brilliance-required” (lower FABs) relative to undergraduates not provided that added information. Our results do not give any evidence for this prediction. Our results, do, however, suggest that this added information dramatically affected undergraduate FAB scores, with markedly different effects for STEM and non-STEM fields. Like survey one, we found no evidence that FAB scores were correlated with female representation in STEM alone for survey two. On the other hand, unlike survey one, FAB scores were positively correlated with female representation in non-STEM alone for survey two (Figures 2, 3; Table 1). This is a reversal of the direction of correlation found in survey one. Notably, this reversal did not happen for STEM fields, where the extra information provided in survey two did not change the pattern of responses with respect to survey one.

### 3.2. Additional Results for Survey One

Our results outlined in the previous subsection were explicitly designed to test the extended FAB hypothesis. The components of the survey and the corresponding results we discuss below were more exploratory than hypothesis-driven, generally designed to explore what conclusions or research directions could be suggested by our data.

To explore which underlying variables can explain patterns in undergraduate FABs, we used exploratory factor analysis (EFA) on undergraduate FAB scores. Two factors were retained, based on parallel analysis, jointly accounting for 65% of the variance (Matsunaga, 2010). The groupings of academic fields that emerged suggested STEM and non-STEM as the latent variables (Table S2). Importantly, contrary to what the FAB hypothesis would predict, female representation varied considerably within each group of fields, suggesting that it is whether a field is STEM or non-STEM, rather than the degree of female representation in a field, that is the important underlying influence on undergraduate FABs.

EFA was implemented using a Varimax rotation and, initially, a four factor solution with the factanal function in R. Parallel analysis indicated that two factors should be retained for survey one and three factors for survey two (Matsunaga, 2010). Using an intermediate cut-off loading score of 0.6, no cross loading occurred. EFA results are similar, with some cross loading for smaller cut-offs, for a wide range of commonly used loading score cut-offs (0.4–0.7) (Matsunaga, 2010).

To assess how personal, educational and demographic characteristics might influence undergraduate FABs, respondents were asked for their major(s) (categorized into STEM, non-STEM or — in the case of STEM/non-STEM double majors — both), their post-college plans (sorted into 19 different categories, i.e., “medical school,” “graduate school for a STEM field,” etc.), GPA, gender, race, class year, and university (Table S1). We coded each university's average ACT score of admitted freshmen as a proxy for institutional prestige. To gauge the effects of these characteristics on undergraduate FAB scores, MANOVA was used with FAB scores as the dependent variables and the respondent characteristics as the independent variables. MANOVA was non-significant (*p* > 0.1 for Pillai's Trace) for all characteristics (Table S4).

As another way to explore how FABs for STEM fields and non-STEM fields differ, we compared mean FAB scores for STEM and non-STEM fields using Welch's *t*-test. For academics, we found no evidence that FABs for STEM and non-STEM fields differed [*t*_(27)_ = –0.87, *p* = 0.390, 95% confidence interval: –0.37, 0.15]. For undergraduates, on the other hand, we found evidence that FABs for STEM and non-STEM fields were different [*t*_(39)_ = –5.61, *p* = < 0.001, 95% confidence interval: –0.85, –0.40], with an average FAB score of 3.61 for STEM and 4.24 for non-STEM fields.

### 3.3. Additional Results for Survey Two

Additional analyses on the second survey produced results similar to those of the first survey. MANOVA using the personal educational and demographic characteristics of respondents was non-significant (*p* >0.1 for Pillai's Trace) across all characteristics (Table S5). Exploratory factor analysis, with three factors retained, cumulatively accounted for 49% of the variance (Matsunaga, 2010). Like the first survey, the groupings of fields that emerge suggested STEM and non-STEM fields as latent variables (Table S3).

### 3.4. Additional Analyses

Insofar as many of our hypotheses are dependent on our Pearson correlations between percent female representation and undergraduate field-specific ability belief scores, we wanted to test whether those correlations are robust to outliers To test this, we conducted a sensitivity analysis. Looking at our regression plots for undergraduate FABs vs. female representation suggests that no academic field was an outlier for STEM fields while Music Composition was an outlier for non-STEM fields (Figure 2). Thus, we re-computed our correlations for non-STEM fields and across all fields, for both versions of the survey, with Music Composition excluded. Our Pearson correlations results were similar with and without the outlier.

Insofar as we used two different survey administration methods (Online and in-class administration), we wanted to test whether, on average, differences in demographic/education variables and/or FAB scores due to the manner in which the survey was presentation (online vs. in-class). To test this, a logistic regression model was fitted separately for each survey version (with gender ratios provided, without gender ratios provided). For each model, the survey presentation type was the outcome and FAB score and demographic/education characteristics were predictors. For both survey versions, we found no evidence of association between survey presentation type and any predictor (*p* > 0.05 for each predictor).

## 4. Discussion

Research on female representation in academia has hitherto been largely focused on explaining female underrepresentation in certain STEM fields. The FAB hypothesis raises the possibility that gender representation in STEM can find a unifying explanation, independent of the STEM/non-STEM divide, in widespread beliefs at play across the entire academic spectrum (Ceci and Williams, 2011; Leslie et al., 2015; Bian et al., 2017). Our results, however, do not support a key prediction of the FAB hypothesis—that undergraduate FABs reflect those of academics. Rather, our results collectively suggest that it is the STEM/non-STEM divide that plays the foremost role in shaping undergraduate FABs.

To whatever extent FABs contribute to the gender gap, this study suggests that undergraduate FABs are not indifferent to the divide, but are instead structured by that divide. We did not find evidence that undergraduate FABs are predictive of female representation within STEM and non-STEM fields separately. Although undergraduate FABs were predictive of female representation across all fields, FABs became an unimportant predictor when the STEM/non-STEM divide was taken into account. Exploratory factor analysis reiterated the importance of the divide, showing it “and not female representation” to be the latent variable influencing undergraduate FABs. Average undergraduate FAB scores differed between STEM and non-STEM fields. We also found no evidence that undergraduate FABs change during college, suggesting the STEM/non-STEM divide consistently shapes undergraduate beliefs, even as they gain prolonged exposure to academics.

The striking difference between the results of our two surveys also reinforces the importance of this divide. While the correlation between undergraduate FABs and female representation remained relatively unchanged for STEM fields, this relationship was reversed for non-STEM: when provided the gender ratio in each field, undergraduates rated the fields with more women as requiring more talent. Our results do not point to a definitive interpretation of this reversal. Our study should, ideally, be unaffected by social desirability bias. But because female representation in STEM is currently a well known and controversial topic, including information about the gender ratio in each field may have evoked social desirability bias. Conservatively, we can say our results suggest that undergraduates are comfortable linking females and intellectual talent in non-STEM fields, and that undergraduate beliefs about the relationship between gender and talent in academia are structured by the STEM/non-STEM divide.

The FAB hypothesis builds on Dweck's work on “growth” vs. “fixed” mindset, proposing that entire academic fields may be placed along a spectrum between the “growth” and “fixed” mindset by measuring the FABs of each field. Our results suggest that academic fields can be fruitfully understood as falling along such a spectrum. Indeed, as Leslie et al. found for academics, we found a considerable spread of undergraduate FAB average scores for academic fields. Furthermore, we found that undergraduate FABs were significantly higher for STEM fields than non-STEM fields. In other words, our results suggest that undergraduates on average view STEM fields as “harder”—requiring more of an unteachable, “fixed” spark of brilliance—than non-STEM fields.

Although it is unclear what could be responsible for the difference between academic FABs and undergraduate FABs, our results raise the possibility that the jump from undergraduate to graduate level could be a critical juncture for shaping FABs. We found no evidence that undergraduate FABs change during college, even as undergraduates accumulate more exposure to academics and, by extension, their FABs. Leslie et al., likewise, found no evidence that the FABs of graduate students, postdoctoral researchers and professors differed (Leslie et al., 2015). This does not rule out the possibility, however, that the US undergraduate experience could be considerably different to that of graduate students and beyond, cultivating considerably different FABs.

Our study is not without limitations. Participants opted in, thus raising the spectre of selection bias. Though our sample includes students from 197 geographically diverse US colleges and universities, the majority came from two universities in Central New York. This may have introduced geographical and socioeconomic biases into our sample. Unlike Leslie et al., who surveyed academics exclusively at “high profile research universities” (Leslie et al., 2015), our responses came from broad range of institutions. When we tested for effects of institutional prestige, however, we found it to be a non-significant explanatory variable. We therefore cannot assess to what extent differences in the FABs of undergraduates and academics might be attributable to the effect of institutional rankings. Universities of widely varied rankings, however, could possibly encourage considerably different beliefs about academic talent.

Many pivotal life decisions that mold gender representation in academia happen at the undergraduate level. Overall, our results suggest that the search for strategies to diversify academic fields should take into account how the STEM/non-STEM divide is central to undergraduate perceptions of both gender and talent in academia.

## Ethics Statement

This study was carried out in accordance with the recommendations of US Department of Health and Human Services regulations for the protection of human participants in research, as followed by the Human Subjects Committee of State University of New York (SUNY) at Oswego, with written informed consent from all subjects. All subjects gave written informed consent in accordance with the Declaration of Helsinki. The protocol was approved by the Human Subjects Committee of SUNY Oswego.

## Author Contributions

KB, DH, and CI: designed research; KB and CI: performed research; KB, SW, AN, and SP: analyzed data; KB, DH, SW, AN, SP, and CI: wrote the paper.

### Conflict of Interest Statement

The authors declare that the research was conducted in the absence of any commercial or financial relationships that could be construed as a potential conflict of interest.
