# Supplementary material for: STEM/Non-STEM Divide Structures Undergraduate Beliefs About Gender and Talent in Academia
Source: Front Sociol. 2019 Apr 12;4:26. doi: 10.3389/fsoc.2019.00026 (PMC8022445; doi:10.3389/fsoc.2019.00026)
Supplement: Supplementary file 1 [file Data_Sheet_1.pdf]

Supplementary Material: STEM/non-STEM Divide Structures Undergraduate Beliefs About Gender and Talent in Academia

1 SUPPLEMENTARY TABLES

Table S1. Survey questions and data coding

Survey question	Question type	Data coding (if applicable)
Please rate the extent to which you believe the below statement is true of each academic discipline listed below: *Being a top scholar in this discipline requires a special aptitude that just cannot be taught.*	Scale	
What is/are your majors?	Open ended	STEM, non-STEM or both (At least one STEM and one non-STEM major)
What is your cumulative GPA?	Open ended	
What is your gender?	Mutiple choice: female, male or other	
What is your race?	Multiple choice: White, African American, American Indian/Alaskan, Asian American, Latino/Hispanic or Middle Eastern	
What are your future plans?	Open ended	medical school, law school, graduate school for non-STEM, graduate school for STEM, graduate school without specified field, professional school for social services, Professional school for health professions, professional school for other professions, professional school for business, job in STEM, job in health professions, job in the arts, job in social services, job in business, job in other professions, job without specified field or unsure
What is your class year?	Multiple choice: freshman, sophomore, junior or senior	As a measure of how long the respondent has been in frequent exposure to academic field-specific ability beliefs, class year was coded quantitatively as 1,2,3 and 4, respectively.
What college/university do you currently attend?	Open ended	As a measure of institutional prestige, university was coded as the average ACT of accepted freshmen for the university.

Table S2. Academic field loadings for exploratory factor analysis of undergraduate survey that excluded gender ratios

Academic field	STEM	Non-STEM	Factor 3	Factor 4
Agricultural Sciences	0.37	**0.62**	0.03	-0.11
Anthropology	0.43	**0.65**	0.06	-0.19
Art History	0.45	**0.60**	0.06	-0.19
Astronomy	**0.68**	0.44	0.14	-0.20
Biochemistry	**0.89**	0.27	0.11	-0.14
Biological/Biomedical Engineering	**0.90**	0.25	0.13	-0.07
Business Management Administration	0.36	0.56	0.12	-0.09
Chemical Engineering	**0.90**	0.27	0.13	-0.06
Chemistry	**0.88**	0.31	0.09	-0.13
Civil Engineering	**0.79**	0.35	0.12	0.02
Classics	0.25	**0.72**	0.16	-0.10
Communication Studies	0.13	**0.70**	0.15	0.01
Computer Science	**0.69**	0.40	0.12	0.16
Earth Science	**0.64**	0.54	0.03	0.08
Economics	0.57	0.59	0.10	0.11
Education	0.22	**0.64**	0.27	0.10
Education Administration	0.23	**0.70**	0.14	0.12
Electrical Engineering	**0.85**	0.29	0.11	0.23
Engineering	**0.86**	0.28	0.15	0.21
English Literature	0.19	**0.75**	0.19	-0.02
Evolutionary Biology	**0.68**	0.52	0.06	-0.06
Foreign Language and Literature	0.35	0.57	0.35	0.21
History	0.30	**0.77**	0.06	0.07
Linguistics	0.41	**0.61**	0.29	0.11
Materials Science Engineering	**0.84**	0.34	0.11	0.14
Mathematics	**0.76**	0.27	0.22	0.14
Mechanical Engineering	**0.88**	0.27	0.13	0.17
Microbiology	**0.85**	0.35	0.12	-0.03
Eastern Studies	0.36	**0.75**	0.08	0.01
Molecular Biology	**0.86**	0.32	0.15	-0.08
Music	0.21	0.40	**0.82**	-0.04
Music Composition	0.29	0.39	**0.78**	0.03
Neuroscience	**0.86**	0.27	0.18	-0.10
Organic Chemistry	**0.88**	0.29	0.11	-0.09
Philosophy	0.20	**0.64**	0.28	0.00
Physics	**0.82**	0.35	0.14	0.00
Political Science	0.38	**0.68**	0.11	0.02
Psychology	0.37	**0.73**	0.04	0.00
Sociology	0.27	**0.79**	0.04	0.03
Spanish	0.30	**0.61**	0.28	0.27
Statistics	**0.63**	0.49	0.07	0.16
**Proportion of variance explained (%)**	**37**	**28**	**5**	**1**

Loading cut-off of 0.6. Loadings that met that cut-off are bolded.

Table S3. Academic field loadings for exploratory factor analysis of undergraduate survey that included gender ratios

Academic field	Non-STEM	STEM	STEM	Factor 4
Agricultural Sciences	0.48	0.39	0.07	-0.01
Anthropology	**0.61**	0.41	0.07	-0.09
Art History	0.48	0.47	0.04	0.03
Astronomy	0.30	0.48	0.26	0.05
Biochemistry	0.15	**0.78**	0.14	0.10
Biological/Biomedical Engineering	0.08	**0.75**	0.24	0.11
Business Management Administration	0.41	0.37	0.12	-0.2
Chemical Engineering	0.07	**0.74**	0.32	0.00
Chemistry	0.10	**0.72**	0.25	0.10
Civil Engineering	0.18	**0.62**	0.37	0.02
Classics	**0.63**	0.20	0.11	0.24
Communication Studies	**0.76**	-0.01	0.06	-0.04
Computer Science	0.24	0.34	0.57	0.11
Earth Science	0.43	0.39	0.36	0.19
Economics	0.39	0.45	0.26	0.10
Education	**0.73**	0.02	0.10	0.06
Education Administration	**0.78**	0.01	0.09	-0.05
Electrical Engineering	0.25	0.42	**0.69**	-0.01
Engineering	0.14	0.48	**0.72**	0.02
English Literature	**0.78**	0.01	0.11	0.12
Evolutionary Biology	0.48	0.58	0.11	0.11
Foreign Language and Literature	0.50	0.27	0.21	0.32
History	**0.67**	0.25	0.12	0.15
Linguistics	0.54	0.28	0.19	0.28
Materials Science Engineering	0.22	0.57	0.52	0.14
Mathematics	-0.03	0.43	0.49	0.34
Mechanical Engineering	0.17	0.52	**0.62**	0.04
Microbiology	0.29	**0.69**	0.16	0.15
Eastern Studies	0.56	0.34	0.07	0.25
Molecular Biology	0.28	**0.64**	0.15	0.22
Music	0.21	0.14	0.00	0.75
Music Composition	-0.09	0.13	0.12	0.74
Neuroscience	0.15	**0.68**	0.17	0.21
Organic Chemistry	0.11	**0.71**	0.23	0.21
Philosophy	0.33	0.26	0.05	0.33
Physics	0.14	0.51	0.42	0.17
Political Science	**0.61**	0.36	0.09	0.02
Psychology	**0.76**	0.16	0.11	-0.03
Sociology	**0.79**	0.13	0.12	-0.01
Spanish	0.58	0.19	0.19	0.31
Statistics	0.46	0.45	0.22	0.16
**Proportion of variance explained (%)**	**21**	**20**	**8**	**5**

Loading cut-off of 0.6. Loadings that met that cut-off are bolded.

Table S4. MANOVA results for the undergraduate survey that excluded gender ratios

Variable	Pillai's Trace	F	df	Error df	*p*
Major	0.38	1.03	126	885	0.41
Future Plans	2.01	0.98	714	5253	0.60
GPA	0.13	1.04	42	293	0.42
Gender	0.38	1.03	126	885	0.41
Race	1.02	1.04	336	2400	0.31
Class Year	0.51	1.43	126	885	0.001
Average ACT	0.13	1.16	42	313	0.71

Type III SS was used.

Table S5. MANOVA results for the undergraduate survey that included gender ratios

Variable	Pillai's Trace	F	df	Error df	*p*
Major	0.38	1.03	126	885	0.41
Future Plans	2.01	0.98	714	5253	0.60
GPA	0.13	1.04	42	293	0.42
Gender	0.38	1.03	126	885	0.41
Race	1.02	1.04	336	2400	0.31
Class Year	0.51	1.43	126	885	0.001
Average ACT	0.13	1.16	42	313	0.71

Type III SS was used.
